# Is systematic training in opioid overdose prevention effective?

**DOI:** 10.1371/journal.pone.0186833

**Published:** 2017-10-31

**Authors:** Albert Espelt, Marina Bosque-Prous, Cinta Folch, Ana Sarasa-Renedo, Xavier Majó, Jordi Casabona, M. Teresa Brugal, REDAN Group

**Affiliations:** 1 Agència de Salut Pública de Barcelona, Plaça Lesseps, Barcelona, Spain; 2 Centros de Investigación Biomédica en Red. Epidemiología y Salud Pública (CIBERESP), Calle Melchor Fernández Almagro, Madrid, Spain; 3 Facultat de Ciències de la Salut de Manresa, Universitat de Vic Universitat Central de Catalunya (UVicUCC), Av. Universitària, Manresa, Spain; 4 Departament de Psicobiologia i Metodologia en Ciències de la Salut, Universitat Autònoma de Barcelona, Campus UAB, Bellaterra, Spain; 5 Centre d'Estudis Epidemiològics sobre les Infeccions de Transmissió Sexual i Sida de Catalunya (CEEISCAT), Agència de Salut Pública de Catalunya (ASPC), Generalitat de Catalunya, Carretera Canyet s/n, Badalona, Spain; 6 Programa de Epidemiología Aplicada y de Campo (PEAC), Instituto de Salud Carlos III, Calle Sinesio Delgado, Madrid, Spain; 7 Subdirecció General de Drogodependències, Departament de Salut de la Generalitat de Catalunya, Carrer de Roc Boronat, Barcelona, Spain; University of Cyprus, CYPRUS

## Abstract

The objectives were to analyze the knowledge about overdose prevention, the use of naloxone, and the number of fatal overdoses after the implementation of Systematic Training in Overdose Prevention (STOOP) program. We conducted a quasi-experimental study, and held face-to-face interviews before (n = 725) and after (n = 722) implementation of systematic training in two different samples of people who injected opioids attending harm reduction centers. We asked participants to list the main causes of overdose and the main actions that should be taken when witnessing an overdose. We created two dependent variables, the number of (a) correct and (b) incorrect answers. The main independent variable was *Study Group*: Intervention Group (IG), Comparison Group (CG), Pre-Intervention Group With Sporadic Training in Overdose Prevention (PREIGS), or Pre-Intervention Group Without Training in Overdose Prevention (PREIGW). The relationship between the dependent and independent variables was assessed using a multivariate Poisson regression analysis. Finally, we conducted an interrupted time series analysis of monthly fatal overdoses before and after the implementation of systematic program during the period 2006–2015. Knowledge of overdose prevention increased after implementing systematic training program. Compared to the PREIGW, the IG gave more correct answers (IRR = 1.40;95%CI:1.33–1.47), and fewer incorrect answers (IRR = 0.33;95%CI:0.25–0.44). Forty percent of people who injected opioids who received a naloxone kit had used the kit in response to an overdose they witnessed. These courses increase knowledge of overdose prevention in people who use opioids, give them the necessary skills to use naloxone, and slightly diminish the number of fatal opioid overdoses in the city of Barcelona.

## Introduction

Most deaths due to illicit drugs are caused by heroin and illicit opioids, with overdose being a leading cause of death among people who use opioids. In 2010, the estimated average EU mortality rate due to overdose among 15-64-year-olds was 18.3 deaths per million inhabitants (7,000–8,000 deaths per year) [1]. Opioid overdose can be fatal or non-fatal, and while fatal overdoses are an important public health problem worldwide, non-fatal overdoses are also important because they cause significant morbidity among victims [2]. While 3–5% of overdoses result in death [3–6], the annual incidence of non-fatal overdose ranges from 9 to 22% [4,7,8]. Opioid overdose can be prevented by taking certain risk factors and risky behaviors into account (e.g. controlling heroin administration route, and avoiding the concomitant use of other drugs) [9], and overdoses can be reversed using simple measures. However, three out of ten people who use opioids in Spain have insufficient knowledge of overdose risk factors or actions to take when witnessing an overdose [10,11].

Training programs in the prevention and management of opioid overdose have proven effective in increasing the relevant knowledge among people who inject Opioids (PWIO) in various settings [12–15], and these programs may be driving the ongoing decrease in overdose mortality [16,17]. In 2009, we designed a generalized Systematic Training in Opioid Overdose Prevention (STOOP) program to be implemented in Catalonia. The program consists in systematic training courses that started in all harm reduction centers in 2009, and was gradually extended to therapeutic communities and treatment centers. By 2013, the STOOP program had already been implemented in all harm reduction centers, treatment centers and therapeutic communities of Catalonia [18]. An specific manual, created to educate and assist in overdose prevention, is the basis for the implementation of STOOP program throughout the territory [18]. The STOOP program is addressed to groups of PWIO and people who use psycho-stimulants, and explain the risks, signs and symptoms of an overdose, and the differences between overdoses caused by opioids and those caused by other psycho-stimulants (see S1 and S2 Tables). This program addresses common myths about dealing with overdoses, and users are instructed on the correct actions to take (i.e. management) when an overdose occurs. Users who have acquired sufficient knowledge (assessed using a test after completing the full program) are given a naloxone kit (two 1 ml bottles of Naloxone (0.4mg/ml), 1 retractable syringe, 1 mask, 2 alcohol wipes, and a brochure with additional information) [18].

To inform future management and policymaking, it is necessary to evaluate the Systematic Training in Opioid Overdose Prevention (STOOP) program. The objectives of this study were to evaluate knowledge about overdose prevention, the use of naloxone, and the number of fatal overdoses following implementation of the STOOP program in Catalonia.

## Material and methods

### Study design and subjects

**Fig 1:**
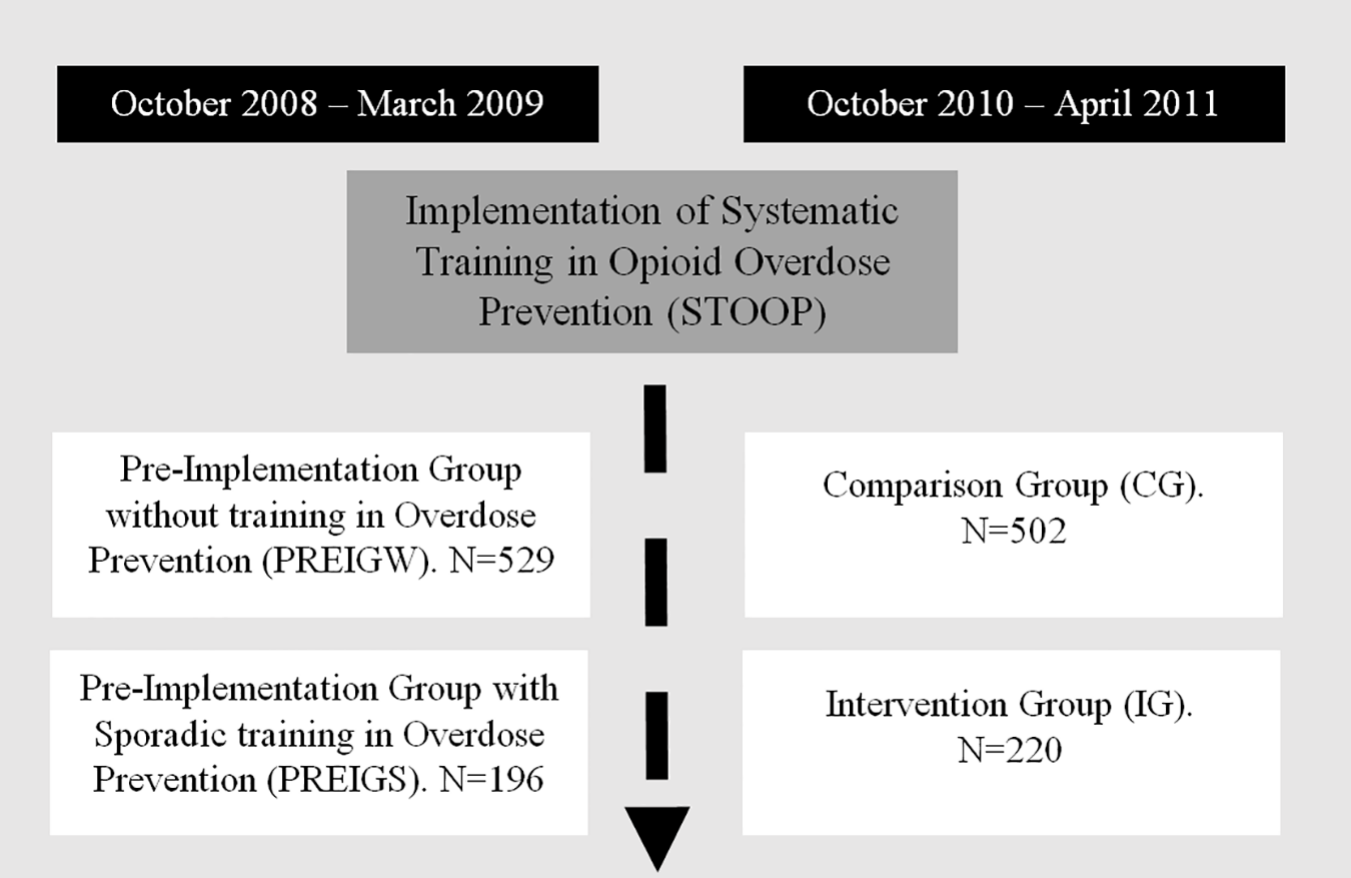
Design of the quasi-experimental study.

We used a quasi-experimental pre-post study design, including a comparison group [19]. The study sample consisted of people who injected opioids and who were attending any of the 18 existing harm reduction centers in Catalonia. Harm reduction centers included needle exchange programs, outreach programs, and supervised injecting facilities. The inclusion criteria were: having injected opioids during the 6 months prior to the interview, and having given written informed consent. Participants were recruited in two distinct periods (each sample was selected independently), before (from October 2008 to March 2009) and after implementation of STOOP program (from October 2010 to April 2011). An independent sample was selected for each period (n_1_ = 725; n_2_ = 722, respectively) [20] (Fig 1). Subjects were assigned to strata in proportion to the volume of visits in each center and the percentage of individuals in each center by country of birth. In centers with less than 5% of foreign-born users, we recruited only native participants. Participants were randomly selected within harm reduction centers.

Finally, we designed an interrupted time series analysis of monthly fatal overdoses in the city of Barcelona (Catalonia) before and after implementation of the STOOP program, including data between 2006 and 2015. The cut-off point was set in January 2013 when the STOOP program had already been implemented in all harm reduction centers, treatment centers and therapeutic communities.

### Data collection

Before and after implementing the STOOP program, trained interviewers conducted face-to-face interviews in each center using an anonymous structured questionnaire adapted from the Itinere project [21] and the World Health Organization [22]. We collected and analyzed data from the following variables associated with knowledge of overdose prevention: age, sex, educational level, irregular income, regular residence, country of birth, lifetime treatment for drug dependency (including drug-free residential treatment or admission to therapeutic communities, in-hospital detoxification, out-patient drug-free treatment, methadone maintenance, and other medication or other treatments), frequency of drug injection, poly-drug use and lifetime history of overdose.

To encourage participation, those respondents participating in the interview before the implementation of STOOP program received 12 Euros and those participating after the implementation of STOOP program received 24 Euros. All the participants provided their written informed consent to participate in this study. The study protocol was approved by a Clinical Ethics Review Board (Hospital Universitari Germans Trias I Pujol, Badalona, Spain).

### Variables

#### Dependent variables

*Knowledge about overdose prevention*: To assess this, we used two open questions, participants were asked to list the main causes of overdose and the main actions that should be taken when witnessing an overdose, as has been done elsewhere [10]. An opioid overdose was defined as an episode that occurred following use of heroin, methadone or other opioids, and is characterized by extreme difficulty in breathing, loss of consciousness and problems waking up or recovering consciousness, and sometimes bluish skin or lips [10]. As described elsewhere [11], the responses to each open question were collected, transcribed verbatim and coded. Responses were then classified in 11 answer categories for causes of overdose (9 correct and 2 incorrect answers) and 15 actions to take when witnessing an opioid overdose (9 correct and 6 incorrect answers) (Table 1). This classification was reviewed and agreed upon separately by three experienced researchers (co-authors MTB, ASR, AE), and a regional working document was used to resolve inconsistencies [23]. From this information, we built two dependent variables based on the number of (1) correct or (2) incorrect responses, revealing participants’ level of knowledge about the causes of overdose, and actions to take when witnessing an overdose.

**Table 1:** Knowledge about overdose prevention: cited causes of overdose, and actions to take when this happens, according to the pre-implementation, comparison, and intervention groups.

	PREIGW	PREIGS	CG	IG
**Causes of opioid overdose**	%	%	%	%
**Correct answers**				
Use of heroin together with other drugs	45.0	66.3	50.8	69.1
Amount injected	66.4	59.2	66.2	59.1
Stronger or purer than usual	15.7	18.4	28.7	32.7
Lower tolerance to heroin	11.7	20.4	16.8	27.7
Change of drug supplier	1.7	1.5	3.6	8.2
Health causes (weakness, predisposition, low defenses. . .)	9.8	10.7	11.1	5.5
Psychological problems / suicide attempt	2.6	2.0	2.0	1.4
Injecting whole dose at once or very quickly	4.3	1.0	1.8	0.9
Intravenous route	5.5	4.1	0.4	0.5
**Incorrect answers**				
Adulterated or cut heroin	23.8	25.5	16.6	13.2
Meaningless and false causes	5.9	4.1	3.6	2.3
**Action to take when witnessing an opioid overdose**	%	%	%	%
**Correct answers**				
First aid	47.8	76.0	80.0	89.0
Call emergency services	59.7	63.8	69.5	72.6
Use of naloxone	0.0	0.0	9.9	43.8
Check consciousness	7.0	15.3	3.1	16.0
Wake up/keep the person awake	12.9	6.6	16.7	7.3
Call police/call for help	6.4	3.1	2.7	3.2
Remove syringe	0.2	1.0	0.6	2.3
Observation	1.9	1.5	3.3	0.9
Facilitate breathing	0.9	0.5	0.6	0.0
**Incorrect answers**				
Shower the person	18.5	17.3	16.0	4.1
Inject substance other than naloxone	11.0	1.5	8.4	2.7
Hit/shake the person	7.6	8.7	6.8	2.3
Meaningless actions	6.0	11.7	5.6	2.3
Make the person move/stand up	14.7	9.2	9.1	1.8
Abandon him/her	0.4	0.0	1.0	0.5

PREIGW: Pre-Implementation Group Without Training in Overdose Prevention; PREIGS: Pre-Implementation Group With Sporadic Training in Overdose Prevention; CG: Comparison Group; IG: Intervention Group

*Number of fatal opioid overdoses per month*. Data on monthly fatal overdoses between 2006 and 2014 came from the register of the Legal Medicine Institute from the city of Barcelona (Catalonia). Overdoses due to other substances or that were suspected to be intentional (suicides) were excluded from the analysis.

#### Independent variables

The main independent variable was *Study Group*: Pre-Implementation Group Without any Courses (PREIGW), Pre-Implementation Group With Sporadic Courses (PREIGS), Comparison Group (CG) or Intervention Group (IG). The PREIGW consisted of all people who injected opioids interviewed between October 2008 and March 2009 who reported that they had not attended any STOOP course (n = 529). The PREIGS consisted of all people who injected opioids interviewed between October 2008 and March 2009 who reported that they had attended some Opioid Prevention Training course (n = 196). The IG (n = 220) consisted of people who injected opioids interviewed between October 2010 and April 2011, when STOOP program had already been implemented and reported that they had attended to an Opioid Overdose Prevention Training course at least once in the previous 2 years. Individuals interviewed after the implementation of the STOOP program who self-reported that they had not participated in any course were included in the CG (n = 502). Before the implementation of the STOOP program there were only sporadic courses with this objective in Catalonia. Thus, existing sporadic courses focused on training a few key PWIO on how to administer naloxone injections to their peers, and were less well prepared and more heterogeneous than the STOOP program.

Finally, to describe and evaluate the effectiveness of naloxone distribution, we separately analyzed the following 4 variables from the IG: having received a naloxone kit in the previous 12 months, having witnessed any overdose in the previous 12 months, having helped a peer suffering an overdose, and having administered the naloxone kit to the peer suffering an overdose.

### Statistical analyses

**Fig 2:**
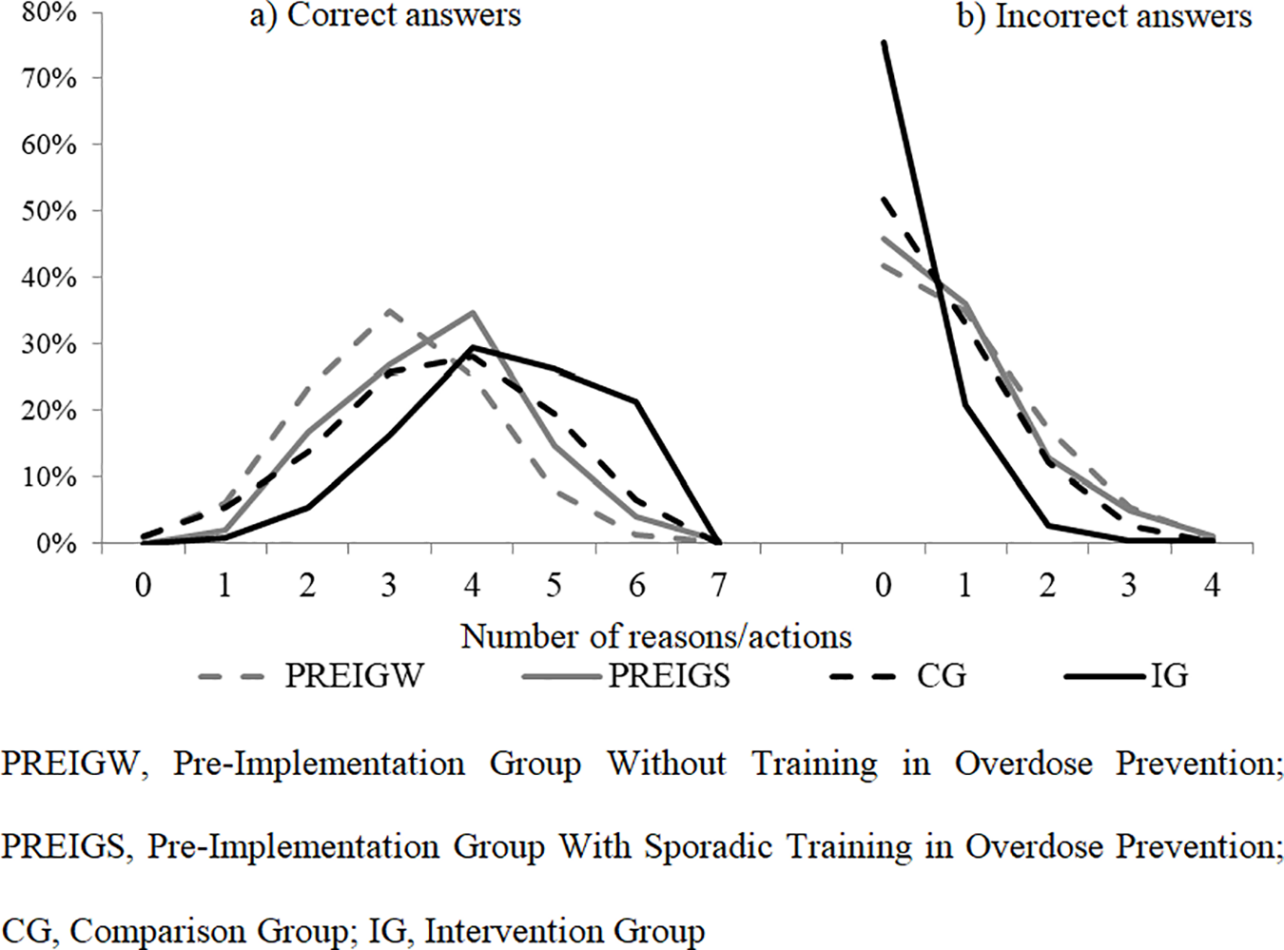
Distribution of people who injected opioids recruited through harm reduction centers in Catalonia according to the number of a) correct and b) incorrect answers about overdose risk factors, and adequate/inadequate actions for reversing or minimizing the effects of an overdose.

To perform an initial pre-post evaluation in terms of curve shifting, for each study group (PREIGW, PREIGS, GC or IG) we plotted the distribution of the number of correct and incorrect answers about causes or actions in overdose prevention and management (Fig 2). Similarly, we assessed users’ level of knowledge by calculating the mean number of correct and incorrect answers about causes or actions, and the corresponding 95% Confidence Intervals (95%CI). To further assess whether the STOOP program resulted in greater knowledge among people who injected opioids, we fit a multivariate Poisson regression model to obtain the adjusted Incidence Rate Ratio (IRR) and 95%CI [STATA syntax: poisson DependentVariable IndependentVariables, vce(robust) irr] [24]. The main independent variable was *Study Group*. The model was adjusted for variables associated with overdose prevention knowledge, as described above [11].

In people from the IG we created a flow chart to summarize the use of naloxone during the year after attending the overdose prevention course.

Finally, we performed a descriptive analysis of the number of fatal opioid overdoses for each month since 2006. To evaluate changes in the number of fatal opioid overdoses after implementation of systematic overdose prevention training courses, we performed an interrupted time-series analysis using quasi-poisson regression models for overdispersed count data, as previously suggested [25]. We compared the number of fatal opioid overdoses per month throughout the time series, controlling for time trend and seasonal patterns using linear trend and including Fourier series terms in the model [26]. [STATA syntax: glm *OverdoseDeath*s *Intervention trend sin1 cos1 sin2 cos2 sin3 cos3 sin4 cos4*, f(poisson) l(log) scale(x2) eform]. We selected 2013 as the intervention year because the STOOP program had not been widely implemented in therapeutic communities and treatment centers before that year [18].

All statistical analyses were performed using STATA 13.0.

## Results

### General characteristics of the sample

Overall, the study included 1,447 people who injected opioids who were attending harm reduction centers in Catalonia. Table 2 shows the between-group differences in the independent variables. These differences were observed for the following variables: age, lifetime drug treatment history, previous overdose history, poly-drug use, irregular income, regular residence at time of interview and country of birth.

**Table 2:** General characteristics of people who injected opioids recruited through harm reduction centers in Catalonia.

	PREIGW (n = 529)	PREIGS (n = 196)	CG (n = 502)	IG (n = 220)	p-value
	%	%	%	%	
**Sex**					
Men	83.7	79.1	84.5	79.5	= 0.19
**Age**					
>30 years	73.1	88.6	75.6	82.3	<0.01
**Educational level**					
Secondary or higher	26.2	20.5	23.1	24.7	= 0.39
**Age at first injection**					
>20	44.5	41.3	44.7	42.2	= 0.81
**Residence**^1^					
Institution or homeless	39.3	41.0	32.5	47.7	<0.01
**Irregular income**^1^					
Yes	60.3	66.2	42.9	51.4	<0.01
**Self-perceived health**^2^					
Poor	38.0	39.0	45.2	39.4	= 0.11
**Time since last drug injection**					
≥30 days	93.6	89.8	91.0	87.3	= 0.04
**Use of Supervised Injecting Facility**^1^					
Less than half of injection days	47.0	50.3	47.6	48.4	= 0.89
**Lifetime drug treatment**					
No	17.6	2.6	21.9	9.1	<0.01
**Poly-drug use**^1^					
≥3 drugs	94.3	98	80.7	83.2	<0.01
**Previous overdose history**					
No	50.9	30.1	44.6	34.5	<0.01
**Country of birth**					
Native (Spain)	53.7	70.9	61.3	61.9	
Eastern Europe	29.3	10.2	25.9	15.1	
Other countries	17.0	18.9	12.8	22.9	<0.01

^1^Previous 6 months

^2^At time of interview; PREIGW, Pre-Implementation Group Without Training in Overdose Prevention; PREIGS, Pre-Implementation Group With Sporadic Training in Overdose Prevention; CG, Comparison Group; IG, Intervention Group.

p-value compares the values for each variable between study groups.

### Effect of attending systematic training in opioid overdose prevention program in knowledge acquisition

In the second round of recruitment, 722 participants were interviewed, of which 30% reported having attended at least one of the STOOP courses (IG). Knowledge about overdose prevention was greater after the implementation of STOOP program. Comparing the number of responses cited by participants (overdose risk factors and the correct actions to take when witnessing an overdose), we found, both in IG and CG, that the distribution curve shifted towards higher scores for correct answers and lower scores for incorrect answers, with the IG showing the most marked curve displacement. In other words, the population who injected opioids as a whole gained knowledge on overdose prevention (Fig 2).

The IG showed the highest levels of knowledge, with a mean number of correct and incorrect answers of 4.4 (95%CI: 4.2–4.5) and 0.3 (95%CI: 0.2–0.4), respectively (Table 3). Table 3 also shows the results of the association between study group and the number of correct and incorrect answers about causes and actions in overdose prevention after adjusting for confounding variables related to knowledge of overdose prevention. Individuals in the IG, CG and PREIGS were more likely to give correct answers reflecting adequate knowledge [IRR 1.40 (95%CI: 1.33–1.47), 1.17 (95%CI: 1.12–1.23) and 1.09 (95%CI: 1.04–1.16), respectively] and less likely to give incorrect answers [IRR 0.33 (95%CI: 0.25–0.44), 0.74 (95%CI: 0.64–0.85) and 0.85 (95%CI: 0.71–1.02)] than those in the PREIGW. The IRR of 1.40 indicates that individuals in the IG gave 40% more correct answers than those in the PREIGW.

**Table 3:** Correct or incorrect answers about causes or actions in overdose prevention among people who injected opioids recruited at harm reduction centers in Catalonia before and after the implementation of Systematic Training in Opioid Overdose Prevention in Catalonia.

	Correct answers					Incorrect answers		
	mean	(95%CI)	aIRR	(95%CI)	mean	(95%CI)	aIRR	(95%CI)
PREIGW	3.1	(3.0–3.2)	1		0.9	(0.8–1.0)	1	
PREIGS	3.6	(3.5–3.7)	1.09	(1.04–1.16)	0.8	(0.7–0.9)	0.85	(0.71–1.02)
CG	3.6	(3.5–3.7)	1.17	(1.12–1.23)	0.7	(0.6–0.7)	0.74	(0.64–0.85)
IG	4.4	(4.2–4.5)	1.40	(1.33–1.47)	0.3	(0.2–0.4)	0.33	(0.25–0.44)

PREIGW: Pre-Implementation Group Without Training in Overdose Prevention; PREIGS: Pre-Implementation Group With Sporadic Training in Overdose Prevention; CG: Comparison Group; IG: Intervention Group. aIRR: Incidence Rate Ratio of correct and incorrect answers before and after the implementation of the STOOP program; aIRR were adjusted for sex, age, educational level, age at first injection, residence, income, self-perceived health, time since last injection in a harm reduction facility, previous treatment for drug dependency, poly-drug use, previous overdose, previous overdose prevention training, and country of birth.

Table 1 shows that people who injected opioids in the IG generally gave more correct answers than those in the CG, PREIGS and PREIGW (e.g. heroin use together with other drugs, use of stronger or purer heroin than usual, reduced tolerance to heroin, and change of drug supplier), and fewer incorrect answers (e.g. taking adulterated or cut heroin). This difference was even greater for actions to take when witnessing an overdose, especially for giving first aid, using naloxone, and checking consciousness. For example, the percentage of responses such as, showering the individual, make them move/stand up, and injecting substances other than naloxone, was lower in the IG than in the other groups (Table 1).

### Implementation of systematic training in opioid overdose prevention and use of naloxone

**Fig 3:**
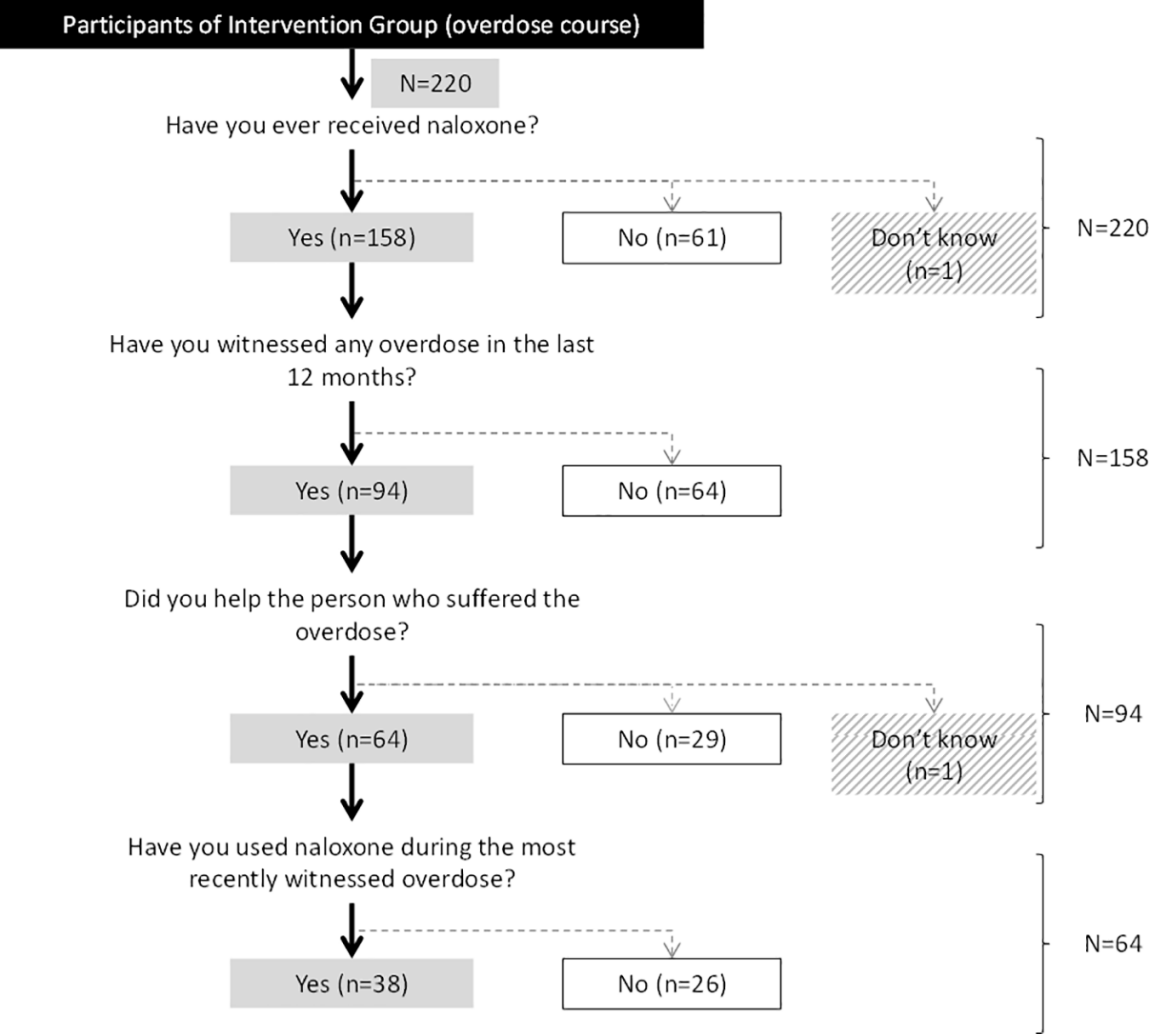
Use of naloxone when witnessing an overdose in the intervention group.

One hundred fifty-eight participants in the IG (72%) received naloxone, of whom 94 (59%) reported having witnessed ≥1 overdoses in the 12 months prior to the interview, 68% of whom (n = 64) had helped the sufferer (59% of these administered naloxone, Fig 3). Thus, 40.4% of people who injected opioids who had received a naloxone kit had used it when witnessing an overdose (Fig 3).

### Implementation of systematic training in opioid overdose prevention courses and fatal opioid overdoses

**Fig 4:**
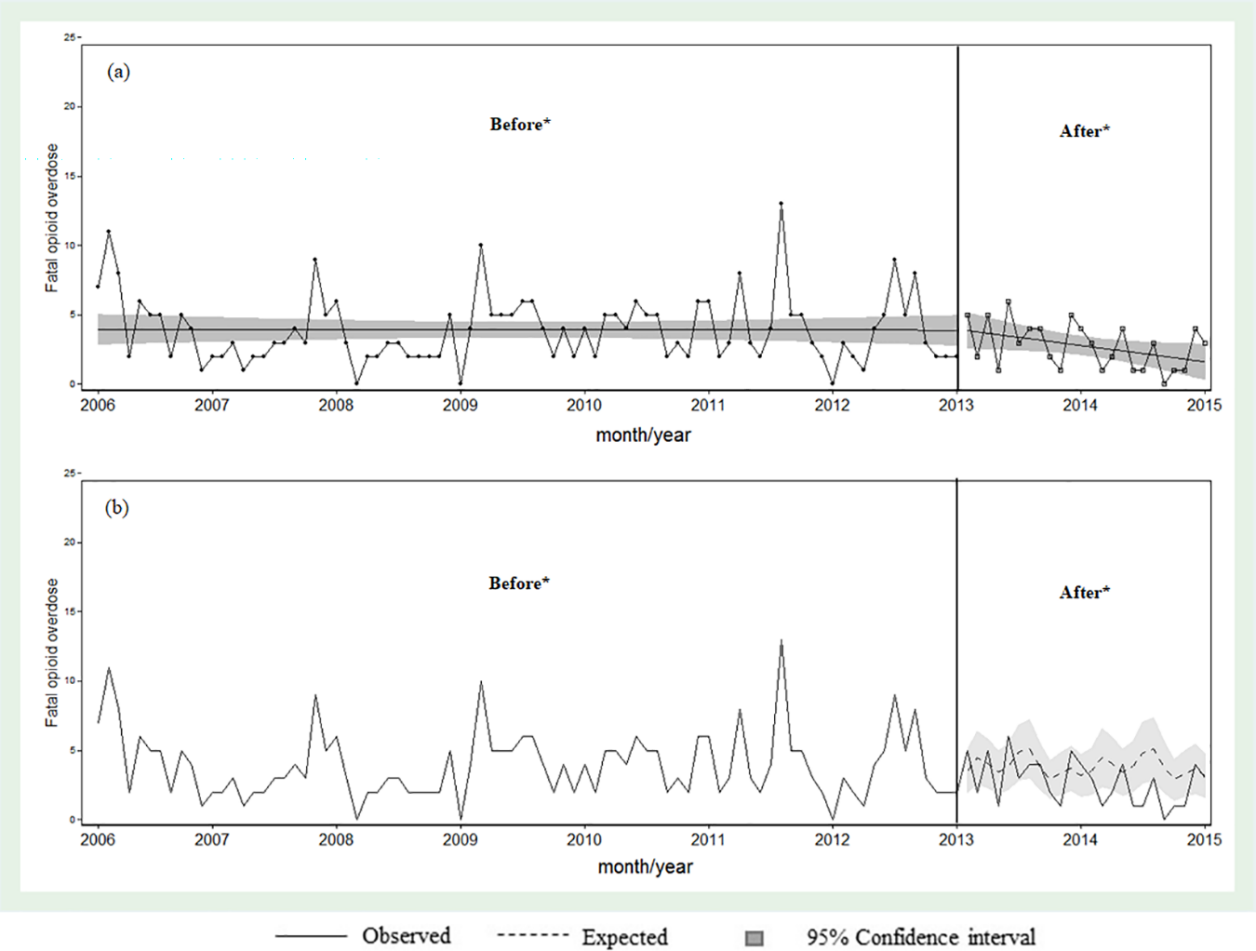
Trend of fatal overdoes (a) and (b) Observed and expected number of fatal opioid overdose per month, Barcelona, 2006–2015.

Fig 4 shows the observed distribution of the number of fatal opioid overdoses since 2006, as well as the distribution (and 95%CI) that would be expected if the STOOP program had not been implemented before and after 2013. The gap between the number of expected and observed fatal opioid overdoses increased over time. In the years 2013 and 2014 there were 27 fewer fatal opioid overdoses than expected if the STOOP program had not been implemented.

## Discussion

Our data indicate that 1) people who use opioids and attended the Systematic Training in Opioid Overdose Prevention (STOOP) program have greater knowledge of overdose prevention than those who did not receive systematic training courses. Our results suggest that these programs improve the general understanding of the population who use opioids as a whole. Individuals who did not attend the STOOP program in 2010–2011 had the same level of knowledge as those who attended sporadic courses in 2008–2009. 2) 59% of trained users that received naloxone had witnessed an overdose in the previous 12 months, and 40% of them had used naloxone during the most recently witnessed overdose. 3) After 2013, there was a decline in the number of fatal overdoses in Barcelona, with 27 fewer fatal opioid overdoses in 2013–2014 than expected if the STOOP program had not been implemented.

We observed greater knowledge in the IG than in the PREIGW, consistent with other studies that found an increase in knowledge [12–14] and better knowledge retention in trained PWID [27,28]. The greater overdose prevention knowledge increased gradually between groups (PREIGW < PREIGC ≤ CG < IG). Thus, STOOP program could result in an overall increase in knowledge among people who use opioids. The increase among untrained users may be due to greater availability of information at harm reduction centers (training courses were held regularly at each center), and greater awareness among staff who had received training on this topic after introduction of STOOP program. This general increase in knowledge could also be due to peer diffusion, in that course participants are known to share what they have learned through their actions and their conversations, both within and beyond the context of overdose events [29]. Peer diffusion of this information is particularly important in this community because of the difficulty in recruiting people who use opioids for training, and the stigma attached to illicit drug use [30]. For this reason, the implementation of the STOOP program was accompanied by specific training on overdose prevention in NGOs, the police department, users’ associations, and social educators involved in municipal plans.

Some external factors independent of the STOOP program (e.g. other campaigns) could have boosted the level of knowledge. However, this is unlikely because the interventions conducted in the population attending harm reduction centers are informed to our organization or supervised by. In addition, the chain of causality between the intervention and the expected outcome was very direct (few intermediate factors) and the study period was relatively short [19,27]. Alternatively, the improvement in knowledge could be due to individual variables related to drug consumption or individual maturation. However, we think this is unlikely because we adjusted the final regression models by drug consumption and individual maturation variables associated with knowledge of opioid overdose prevention. In addition, improvements in knowledge about some causes or actions in overdose prevention were clearly related to attendance at the courses, e.g. an increased number of correct answers about first aid, calling the emergency services and using naloxone, and a decreased number of incorrect answers such as making the victim take a shower or move/stand up (Table 1).

We found that 40.4% of people who use opioids who attended a STOOP program and received a naloxone kit had used it during the last overdose they witnessed, a similar proportion to that observed in San Francisco (40%) [13] and in New York (58%)[31]. A meta-analysis done in 2015 [32], found that 9% of naloxone kits distributed to trained users will have been administered to a peer within the three months of supply. This is consistent with effective knowledge retention [27], and greater ability to recognize an overdose and act appropriately after training [33]. For example, it has been shown that PWIO with increased knowledge can administer naloxone to an overdose victim and prevent a fatal overdose [34]. However, some trained PWIO did not use their naloxone kit during the last overdose they witnessed. Previous studies have not been able to clarify the reasons for this because of small sample sizes, although the reasons given include loss of the naloxone kit, that the witness was no longer using drugs, and that the victim was already dead when found [27,35]. Tobin and colleagues [13] suggested that naloxone kits were generally not lost, stolen or confiscated, although in our study 16% of participants in the overdose prevention program who received a naloxone kit reported that they were not carrying it when they witnessed an overdose (results not shown). Another important issue is what could happen in countries with a different justice approach to drug use. Thus, people who use opioids need police permission to carry naloxone in public, which in turn requires fluid communication and agreement between the police and the public administration. Therefore, after the STOOP program, participants receive a card identifying them as experts in overdose prevention.

Finally, we observed a slight decline in the number of overdose deaths in Barcelona after 2013, which could be related to the STOOP program. In June of 2012 only 43.5% of PWIO in Catalonia had participated in an overdose prevention course, with different percentages among individuals undergoing treatment (32.1%), those in therapeutic communities (35.2%), and those using harm reduction centers (66.7%) [36]. While we cannot directly attribute this reduction to the implementation of STOOP program, our results are consistent those reported in Scotland [37] and the USA [17]. Implementation of Scotland’s National Naloxone Programme was associated with a 36% decrease in the proportion of opioid-related deaths during the four weeks after release from prison. The results on greater knowledge of overdose prevention are encouraging because greater knowledge could be related to reduced overdose risk [38,39]. Moreover, a systematic review found that the Take-Home-Naloxone provision reduced fatal overdoses among program participants themselves, and also among fellow people who use opioids and the wider community, and significantly reduced overdose mortality with respect to communities without implementation [34].

### Strengths and limitations

Finally, we note some strengths and weaknesses of our study. This is a study that uses an extended and representative sample of people who inject opioids and use harm reduction centers. The dependent variable, knowledge of overdose prevention, while not as straightforward as others [40], was asked in exactly the same way in each wave of this study, and the answers were classified in the same way, with broad consensus among the study researchers, as explained elsewhere [11].

In terms of limitations, we could not differentiate between knowledge acquisition, retention and application. Although we could not control for the time between attending the systematic courses and the study interview, we know that they were not done at the same time. The STOOP program started in the third trimester of 2009 and the first surveys started in October 2010. Moreover, these courses are systematically addressed to all users, and reminders are issued and re-training offered a year after the first course. In this sense, the course remains active all year. Finally, we could not observe the direct impact of knowledge acquisition on some preventive practices, although we observed a slightly decline in the number of fatal overdoses in the city of Barcelona.

## Conclusions

We found that Systematic Training in Opioid Overdose Prevention (STOOP) program increases knowledge among people who injected opioids. In addition, a high percentage of trained people who injected opioids (40%) used naloxone during the last overdose they witnessed. The STOOP program should also be deployed in prisons, since PWID have a higher risk of a drug-related death once they are released [41,42]. However, further research is required to investigate and develop new strategies to increase the use of naloxone where necessary.

## Supporting information

S1 Table Short Guide on counselling for heroin and cocaine overdose. (DOCX)

S2 Table Key points for training users in the prevention and treatment of overdoses. (DOCX)

S1 File This is the S1 File Dataset for Tables 1, 2 and 3. (DTA)

S2 File This is the S2 File Dataset for Fig 3. (DTA)
