# Supplementary material for: Is systematic training in opioid overdose prevention effective?
Source: PLoS One. 2017 Oct 31;12(10):e0186833. doi: 10.1371/journal.pone.0186833 (PMC5663400; doi:10.1371/journal.pone.0186833)
Supplement: S1 Table — (DOCX) [file pone.0186833.s001.docx]

**S1 Table.** **Short Guide on counselling for heroin and cocaine overdose.**

| Heroin |
| --- |
| **RISKS of suffering an overdose** |
| Consuming the usual quantity after break in consumption |
| Mixing substances such as heroin, methadone, benzodiazepines and alcohol. |
| Consumption via injection |
| **IDENTIFYING an overdose** |
| Does not respond to stimulation or is unconscious |
| Slow breathing or not breathing |
| Small pupils |
| Lips and fingers blue |
| **ACTION** |
| **Protect and protect yourself** |
| Remove dangerous objects (needles, glass, etc.) from the area surrounding the person who is overdosing |
| **Alert** |
| It is very important to **call 112** |
| Say whether the person is unconscious or not breathing |
| Clearly indicate your location |
| **Aid** |
| Inject intramuscular Naloxone into the shoulder |
| If they are sleeping and breathing, place them in the recovery position |
| If they are not breathing and do not have a pulse, begin cardiopulmonary respiration |
| **Wait** |
| Stay with the victim until the ambulance arrives |

More information: <http://drogues.gencat.cat/web/.content/minisite/drogues/noticies/actualitat/arxiu/Espelt-et-al_2015.pdf>
