# Supplementary material for: Is systematic training in opioid overdose prevention effective?
Source: PLoS One. 2017 Oct 31;12(10):e0186833. doi: 10.1371/journal.pone.0186833 (PMC5663400; doi:10.1371/journal.pone.0186833)
Supplement: S2 Table — (DOCX) [file pone.0186833.s002.docx]

**S2 Table.** **Key points for training users in the prevention and treatment of an overdose.**

**1.** Administer informed consent forms. **General Evaluation** of the program, which should be carried out before beginning the 1^st^ (*pre* questionnaire) and 2^nd^ workshop (6 months) (*post* questionnaire).

Between the questionnaire and the beginning, read the **workshop rules** (attendance at the entire workshop, no leaving before the end, etc.), which the user must sign.

**2. Risks** of suffering an overdose and measures to prevent it.

**3. Signs and symptoms** of overdose; differences between overdose by opioids and psychostimulants.

**4.** **Myths** about what to do in case of an overdose.

**5.** How to **act** when faced with an overdose.

Role-playing game

Recovery position with participants and cardiopulmonary resuscitation (CPR) using a mannequin.

**6.** Present the naloxone kit and its **components. Propose a role-playing game on how to open the naloxone correctly without cutting yourself and how to load the syringe, IT’S SCREWED ON! (it is not necessary to open it, just to explain it while demonstrating). Also, demonstrate how to put the facemask on (this side up). IT IS IMPORTANT TO PREVENT INFECTIOUS DISEASES.**

**7.** Evaluate the knowledge acquired.

**8.** Give the **Overdose Health Agent** (ASSO) card to users who pass this evaluation.

**9.** Give €5 in the 1^st^ workshop and €10 in the 2^nd^.

**10.** If the user has passed the evaluation, give them a kit.

**11.** After 6 months, perform the group follow-up workshop; administer the follow-up questionnaire (*post* questionnaire) beforehand.

**12.** After one year, renew the ASSO card:

*automatic renewal if you know that that the user has the required knowledge;

*if in doubt, administer the knowledge evaluation questionnaire, and if the result is positive, give them the card;

More information: <http://drogues.gencat.cat/web/.content/minisite/drogues/noticies/actualitat/arxiu/Espelt-et-al_2015.pdf>
